# Myosin and gelsolin cooperate in actin filament severing and actomyosin motor activity

**DOI:** 10.1074/jbc.RA120.015863

**Published:** 2020-12-17

**Authors:** Venukumar Vemula, Tamás Huber, Marko Ušaj, Beáta Bugyi, Alf Månsson

**Affiliations:** 1 Department of Chemistry and Biomedical Sciences, Linnaeus University, Kalmar, Sweden; 2 Department of Biophysics, Medical School, University of Pécs, Pécs, Hungary

**Keywords:** actin, myosin, gelsolin, dynamics, cooperativity, Ca^2+^-dependence, severing, *in vitro* motility assay, actin-binding proteins, ABP, actin-binding protein, COT, cyclooctatetraene, HMM, heavy meromyosin, LISS, low ionic strength solution, NBA, 2 mM 4-Nitrobenzyl alcohol, TIRF, total internal reflection fluorescence, TMCS, trimethylchlorosilane

## Abstract

Actin is a major intracellular protein with key functions in cellular motility, signaling, and structural rearrangements. Its dynamic behavior, such as polymerization and depolymerization of actin filaments in response to intracellular and extracellular cues, is regulated by an abundance of actin binding proteins. Out of these, gelsolin is one of the most potent for filament severing. However, myosin motor activity also fragments actin filaments through motor-induced forces, suggesting that these two proteins could cooperate to regulate filament dynamics and motility. To test this idea, we used an *in vitro* motility assay, where actin filaments are propelled by surface-adsorbed heavy meromyosin (HMM) motor fragments. This allows studies of both motility and filament dynamics using isolated proteins. Gelsolin, at both nanomolar and micromolar Ca^2+^ concentration, appreciably enhanced actin filament severing caused by HMM-induced forces at 1 mM MgATP, an effect that was increased at higher HMM motor density. This finding is consistent with cooperativity between actin filament severing by myosin-induced forces and by gelsolin. We also observed reduced sliding velocity of the HMM-propelled filaments in the presence of gelsolin, providing further support of myosin-gelsolin cooperativity. Total internal reflection fluorescence microscopy–based single molecule studies corroborated that the velocity reduction was a direct effect of gelsolin binding to the filament and revealed different filament severing pattern of stationary and HMM propelled filaments. Overall, the results corroborate cooperative effects between gelsolin-induced alterations in the actin filaments and changes due to myosin motor activity leading to enhanced F-actin severing of possible physiological relevance.

Actin is a major cytoskeletal protein, constituting 5 to 10% of the cellular protein content in eukaryotes (1, 2). It has vital roles not only in muscle contraction and nonmuscle cell motility but also in a variety of other cell functions such as intracellular transport, cytokinesis, membrane dynamics, cell signaling, and regulation of cell–cell contacts (3). In muscle contraction and several other processes, actin filaments (F-actin) interact with the molecular motor myosin II to produce force and motion by sliding of actin and myosin relative to each other. Other functional roles of actin rely on its dynamic properties with polymerization and depolymerization regulated by a range of actin-binding proteins (ABPs) and varying cellular conditions (4, 5, 6, 7).

Gelsolin is one of the most abundant and potent actin filament severing, capping, and nucleating proteins (8, 9, 10, 11, 12, 13) among the plethora of ABPs (14, 15) that govern remodeling of the actin cytoskeleton in response to various cues (8). The gelsolin activity is regulated by the complex interplay between calcium (Ca^2+^), polyphosphoinositide 4, 5-bisphosphate and ATP (16), but it is also partially activated by low pH (17) and high temperature (30–40 °C) (18, 19). Binding of Ca^2+^ to several conserved sites on gelsolin, with Ca^2+^ affinities ranging from the 100 nM to 10 μM, changes gelsolin structure to facilitate binding to actin and subsequent actin filament severing (11, 20) by weakening the noncovalent bonds between actin subunits (21). After severing, gelsolin remains attached to the barbed end (fast polymerizing, plus end) of the filament, thus blocking addition of actin monomers (G-actin). However, gelsolin can also nucleate polymerization by binding to two G-actin units *in vitro* (19).

The *in vitro* motility assay is a frequently used tool in studying the motion generated by the interaction between myosin and actin, powered by turnover of MgATP (22, 23). Generally, myosin motor fragments such as heavy meromyosin (HMM) are adsorbed to modified glass surfaces in a controlled environment while the propulsion of fluorescently labeled actin filaments is observed using microscopy. The speed of the actin filament movement (sliding velocity) depends on several factors (22, 24), such as the density of myosin heads (HMM), MgATP concentration, ionic strength, pH, temperature, and the surface modification used for HMM adsorption. A lowered HMM surface density leads not only to reduced actin filament sliding velocity but also to a reduced filament fragmentation during the HMM propelled sliding (25). Such filament fragmentation is the basis for an increased number of filaments and reduced average length with time in the *in vitro* motility assay. However, importantly, actin filament fragmentation due to myosin motor activity may also have critical roles in cellular physiology (7, 26, 27, 28) either alone or in cooperation with other ABPs, *e.g.*, as proposed for cofilin (26, 29).

In accordance with evidence that actin filaments exhibit intrinsic structural polymorphism in response to varying environmental conditions (30, 31, 32), structural transitions along F-actin induced by ABPs are believed to be important for cooperation with fragmentation that is due to myosin motor activity (27, 33). Thus, several studies suggest that binding of a range of ABPs allosterically produce long-range structural transitions along the actin filament (34, 35, 36, 37, 38, 39, 40, 41, 42, 43). For instance, gelsolin binding to an actin filament has been found to produce changes of this type (27, 44, 45), which may be expected to change the mode of actin binding of other proteins including myosin. Furthermore, one may consider the possibility of cooperativity between the filament severing induced by gelsolin binding on the one hand and myosin motor activity in the presence of MgATP on the other hand (44, 45). However, to the best of our knowledge, these issues have not been previously studied.

Here, we therefore investigated the effect of gelsolin on HMM-induced actin filament sliding in the *in vitro* motility assay to assess possible gelsolin-induced changes in the myosin binding to actin or in the subsequent generation of motion. We also studied gelsolin-mediated F-actin severing during HMM propelled actin filament sliding and when actin filaments were bound to surface-immobilized HMM in the absence of motility. The experiments were performed either at a low [Ca^2+^] where little activation of actin binding and severing by gelsolin is expected (46, 47) or at sufficiently high [Ca^2+^] expected to fully activate gelsolin (47). Experiments were further performed at different HMM surface densities and using total internal reflection fluorescence (TIRF)–based single molecule observation of the gelsolin binding and severing pattern along actin. The results provide evidence for cooperative effects between HMM and gelsolin both in actin filament severing and in actomyosin motor function. The molecular mechanisms and the relevance of these findings in cellular function are discussed.

## Results

### Gelsolin accelerates actin depolymerization in bulk assays

The actin severing mechanism of gelsolin is complex. A Ca^2+^ concentration in the micromolar range (∼0.1–5 μM; pCa 5.3–7) is typically required to modify gelsolin to its fully active conformation (closed to open) that is competent for F-actin severing and barbed end capping (21, 48, 49, 50). However, other experimental conditions (*e.g.*, concentration of reagents used in the assay in addition to Ca^2+^, such as gelsolin itself, actin, myosin, and ATP) may influence the gelsolin binding to actin filaments and the gelsolin-mediated filament splitting rates.

**Figure 1:**
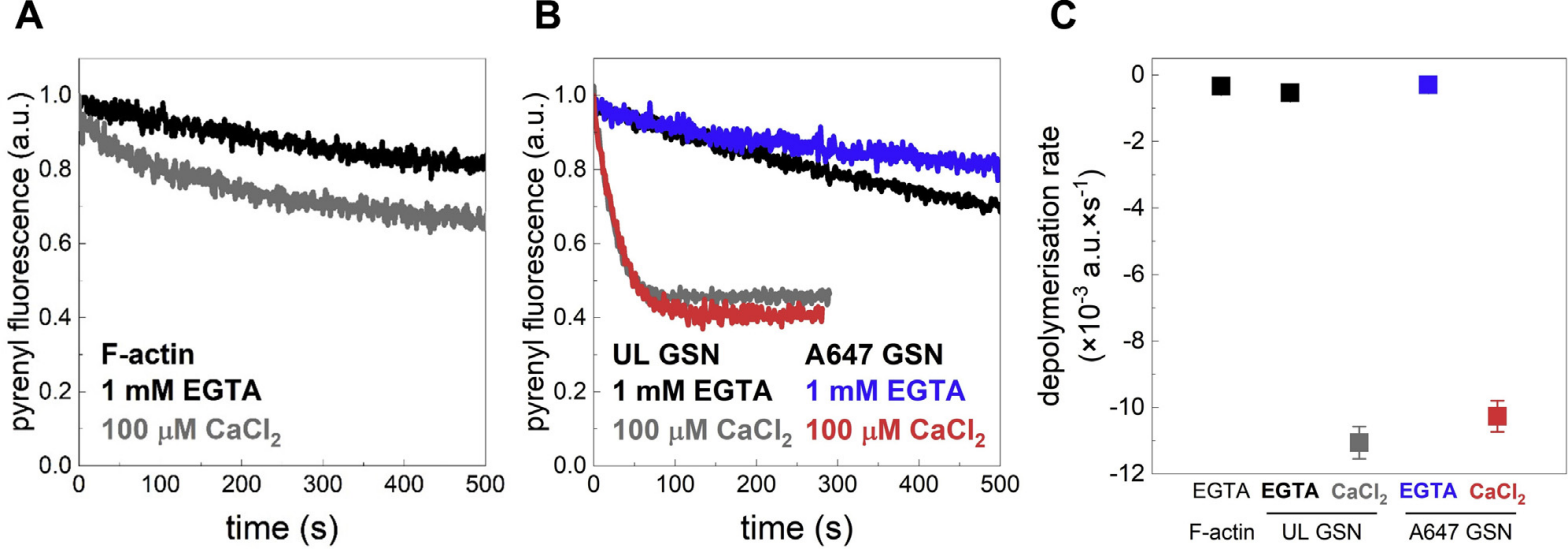
**Ca**^**2+**^**enhances the actin filament severing activity of gelsolin.** For the depolymerization assays, actin was polymerized and diluted in polymerization buffer to a final concentration of 20 nM to induce spontaneous depolymerization. Gelsolin (GSN) was used at 5 nM final concentration at pCa 4.0 or pCa > 9 (1 mM EGTA added). *A*, spontaneous actin disassembly in the presence of either 100 μM CaCl_2_ (pCa 4.0) or 1 mM EGTA (pCa > 9) but in the absence of GSN. Data are presented as the average of two independent measurements at each condition. *B*, actin disassembly in the presence of gelsolin (5 nM) (unlabeled; UL or Alexa-647 labeled; A647) and in the presence of either 100 μM CaCl_2_ (pCa 4.0) or 1 mM EGTA (pCa > 9). Data are presented as the average of 2 (A647 GSN) and 4 (UL GSN) independent measurements. *C*, depolymerization rate (negative) derived from linear fitting of the pyrenyl transients. Mean ± SD, n = 2 to 4. Note negligible effects of gelsolin on depolymerization rate in the absence of Ca^2+^ but a substantial accelerating effect with added Ca^2+^ (pCa 4.0).

To investigate the basal F-actin severing activity of the gelsolin preparations used in the present study under standardized conditions, we first checked the disassembly of actin filaments in solution in the presence of EGTA (1 mM) or CaCl_2_ (100 μM) (Fig. 1). These conditions correspond to pCa>9 and pCa 4.0, respectively. We used F-actin formed upon polymerization of 50% pyrene-labeled G-actin followed by dilution to a final concentration of 20 nM, below the barbed end critical concentration. These conditions favor the monitoring of spontaneous monomer dissociation. Filament disassembly kinetics was monitored by recording the pyrenyl emission as a function of time. In a first set of experiments, we found that the rate of spontaneous actin depolymerization is very low in the presence of EGTA, whereas addition of CaCl_2_ slightly favors the monomeric form of actin even in the absence of gelsolin (Fig. 1*A*). This may be attributed to the effects of the divalent cation on the mechanical properties of F-actin (51, 52).

Next, we investigated the F-actin severing activity of gelsolin using two different gelsolin batches, where gelsolin was either unlabeled or labeled with Alexa-647 (Fig. 1*B*). The results from these assays, summarized in Figure 1*C*, demonstrate that in the absence of Ca^2+^ (1 mM EGTA), actin disassembly was not significantly influenced by addition of gelsolin (5 nM) at shorter timescales (<200 s) (Fig. 1, *B*–*C*). However, a slightly faster (∼1.6-fold) rate than that for spontaneous disassembly was detected in the presence of unlabeled gelsolin as compared with the labeled protein, an effect that became apparent at longer times. As expected, the disassembly rate was appreciably increased (∼20–35-fold) upon changing to excess CaCl_2_ (pCa 4.0) whether unlabeled or Alexa-647-labeled gelsolin was used (Fig. 1, *B*–*C*). Thus, the disassembly rates both in the absence and in the excess of Ca^2+^ were similar for Alexa-647-labeled and unlabeled gelsolin, and both proteins showed strong calcium dependent severing activity.

### Cooperativity between gelsolin-mediated and myosin-mediated effects on actin filaments in the *in vitro* motility assay

To the best of our knowledge, the severing activity of gelsolin during an *in vitro* motility assay has not previously been reported. Here, we performed studies using this assay to elucidate the interactions between gelsolin-mediated F-actin severing and actin–myosin motor activity.

**Figure 2:**
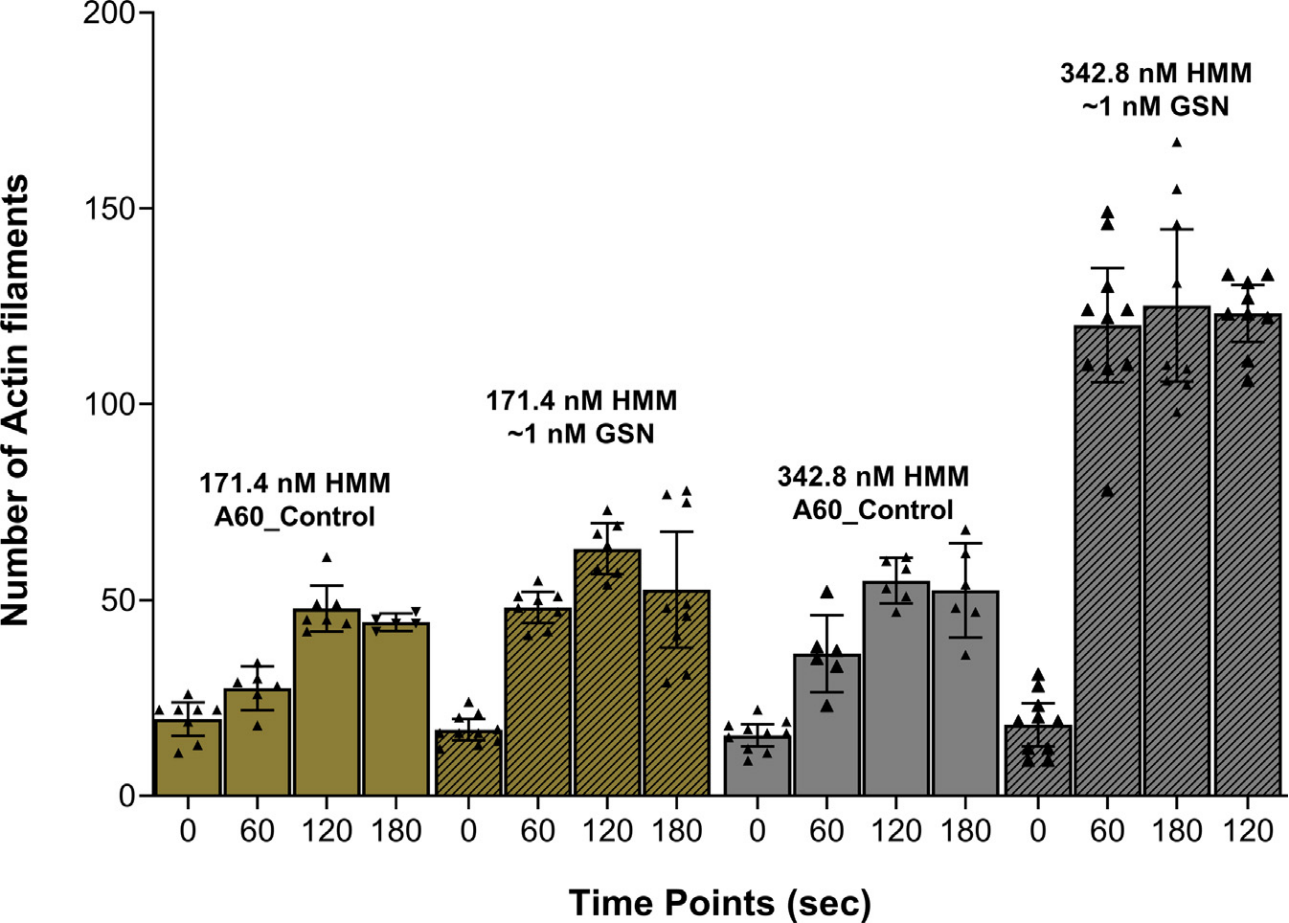
**Number of actin filaments in the *in vitro* motility assay at different time points after addition of MgATP-containing assay solution (to start motility) including Ca**^**2+**^**(pCa 5.7) without or with gelsolin (GSN; ∼1 nM) and different surface incubation concentrations of HMM (171.4 or 342.8 nM).** Concentration ratio of added actin and gelsolin, [Actin(monomer)]/[gelsolin]: 5. Data shown as mean ± 95% CI. Temperature: 25 to 27 °C. Data from individual experiments superimposed on bars representing mean values. Nonoverlapping 95% CIs between bars indicate (approximate) statistical significance for differences corresponding to *p* < 0.05. HMM, heavy meromyosin.

First, we noted (Fig. S1, *A*–*B*) that at 5 nM gelsolin in the assay solution, the actin filaments were fragmented quite rapidly even at low [Ca^2+^] (pCa 8.2). If the Ca^2+^ concentration was increased to pCa 7.2 (Fig. S1*C*) and higher (tested for pCa 5.7 in Fig. S1*D*), the severing rate increased appreciably, leaving virtually no observable filaments left 30 s after initiation of the motility assay. We attribute this effect to the rapid severing and disassembly of F-actin (53, 54) (*cf.* Fig. 1). We next tested gelsolin in the nanomolar range (0.75–1 nM) (55) using assay solution A60 (see Experimental Procedures) without (pCa>9) and with added Ca^2+^ (pCa 5.7) with a concentration of F-actin corresponding to a subunit concentrations of ∼5 nM. First, we noted that the number of filaments in the *in vitro* motility assay increased with time even in the absence of gelsolin because of fragmentation by myosin-induced forces (Fig. 2, A60_control). However, we also noted that the rate of severing at 1 nM gelsolin was sufficiently slow to allow microscopy-based quantitative analysis of the severing rate.

At the lowest HMM surface density tested (HMM incubation concentration 171.4 nM), we observed an approximately two-fold early increase (0–60 s) in the number of actin filaments in the *in vitro* motility assay in the presence of gelsolin as compared with its absence (Fig. 2). The HMM incubation concentration of 171.4 nM is expected to give less than saturating density of HMM on the motility assay surface (*e.g.*, (56, 57); supplemental information of latter paper). Remarkably, with a higher HMM incubation concentration (342.8 nM), expected to saturate the surface (56), there was a 5- to 7-fold increase in the number of actin filaments within 60 s in the presence of gelsolin as compared with control samples (Fig. 2). The increase in the filament number with time was significantly higher than in the absence of gelsolin as indicated by nonoverlapping 95% confidence intervals. It should also be noted from the data in Figure 2 that in these experiments, increased HMM surface density had negligible effects on the motor induced severing in the absence of gelsolin. The marked difference between the effects of increased HMM density on motor induced severing in the presence and absence of gelsolin (with minimal effects of gelsolin at the lowest motor density) suggests cooperative effects between gelsolin induced severing and motor induced severing.

**Figure 3:**
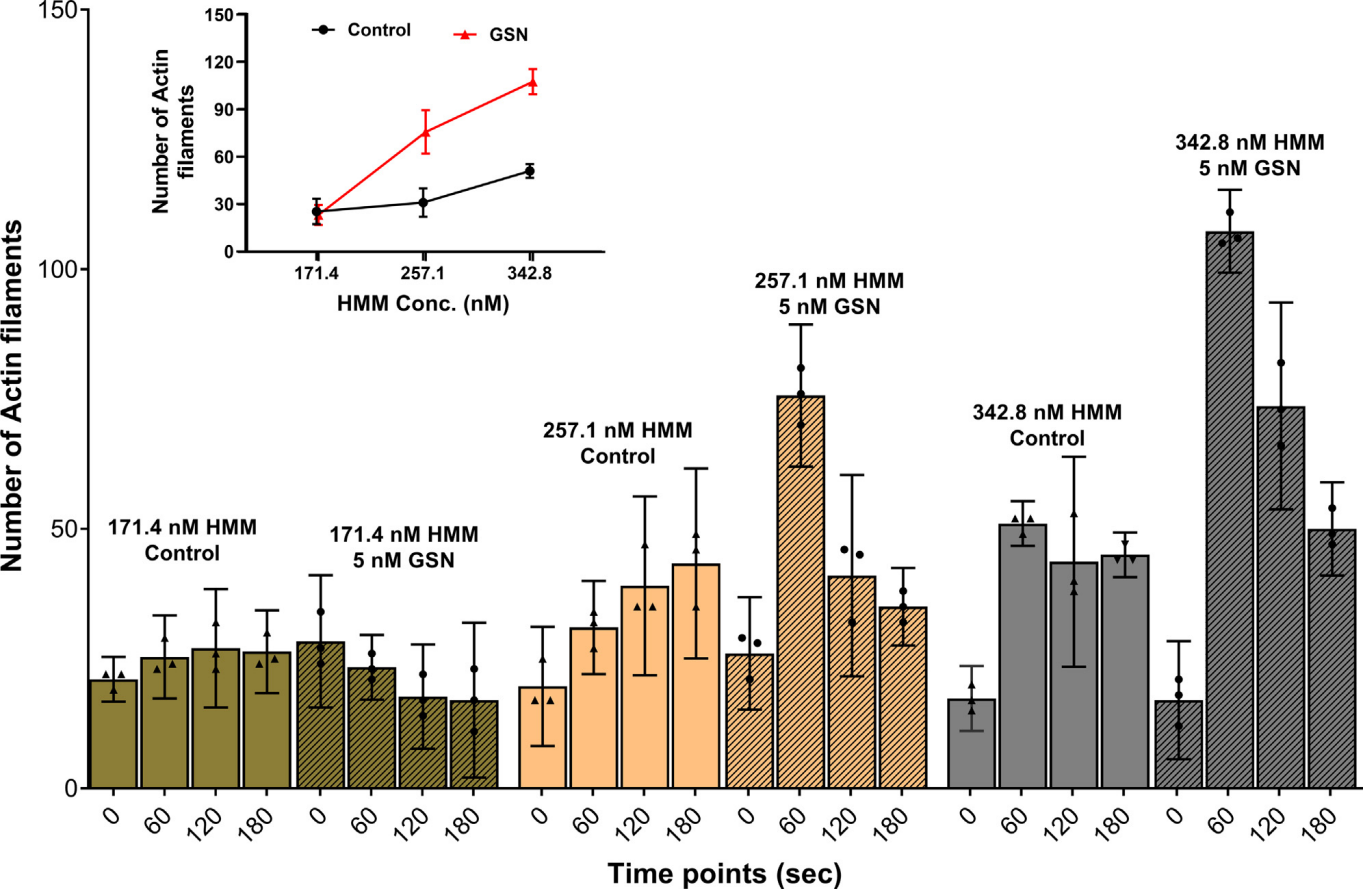
**Number of actin filaments in the *in vitro* motility assay at different time points after addition of MgATP (to start HMM propelled actin sliding) under conditions with preincubation (2 min) of the motility assay flow cell with HMM at different concentrations; 171.4 to 342.8 nM to give increasing HMM surface density.** Motility assays conditions with Ca^2+^(pCa 8.2) with or without gelsolin (GSN; 5 nM). [Actin(monomer)]/[gelsolin]: ∼1. Data shown as mean ± 95% CI. Nonoverlapping 95% CIs indicate statistically significant differences corresponding to *p* < 0.05. Temperature: 25 to 27 °C. Data from individual experiments superimposed on bars representing mean values. Note, we attribute the decrease in the number of filaments at late time points to detachment of filaments from the surface and difficulty to observe short filaments because of weak total fluorescence (see further text). Inset: number of actin filaments in the *in vitro* motility assay 60 s after MgATP addition as a function of the HMM concentration used for preincubation of the flow cell. CI, confidence interval; HMM, heavy meromyosin.

We also studied the effects of varying HMM density on gelsolin-mediated fragmentation at low [Ca^2+^] (pCa 8.2) in Figure 3 as compared with the high [Ca^2+^] (pCa 5.7) used in Figure 2. The use of low [Ca^2+^] (Fig. 3) allowed us to increase the gelsolin concentration to 5 nM without filament severing being too fast for microscopy-based quantification. An increased actin filament fragmentation rate is observed for increased HMM incubation concentration in the range 171.4 to 342.8 nM. Importantly, similar to the situation at 1 nM gelsolin and high [Ca^2+^] (Fig. 2), the number of filaments increased to greater extent with time (from 0 to 60 s) in the presence than in the absence of gelsolin (5 nM) at low [Ca^2+^] (Fig. 3). However, the effect of gelsolin was only observed at the two highest HMM concentrations used. This is clearly seen from a summary of the results corresponding to the 60 s time points as shown in the inset of Figure 3. The findings in Figure 3 suggest that gelsolin binds to the actin filaments also at low [Ca^2+^] ([pCa 8.2], see further below). The results further suggest that gelsolin bound to actin at low [Ca^2+^] potentiates fragmentation of the filaments because of the forces produced by myosin motor activity. To summarize, the results in Figure 3 suggest that, also at low [Ca^2+^], gelsolin addition noticeably increases the HMM-induced actin filament severing, at least at the highest HMM densities studied. These findings are consistent with cooperative effects between motor-induced and gelsolin-induced severing also at low [Ca^2+^].

**Figure 4:**
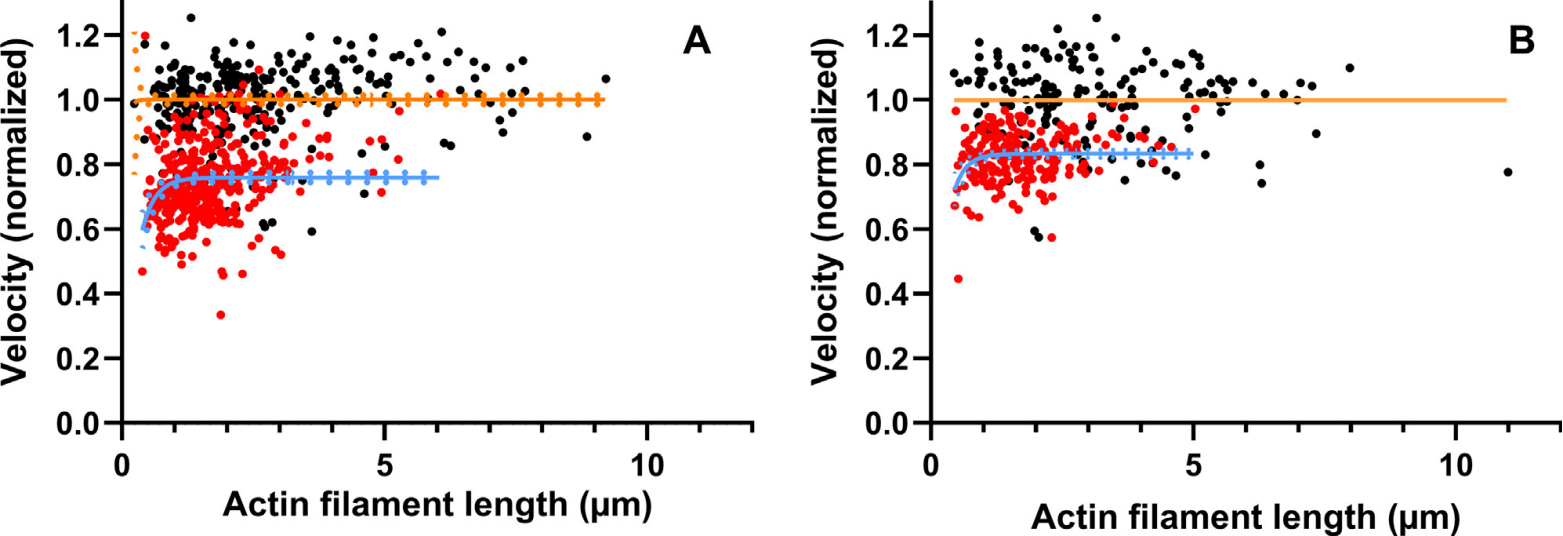
**Effects of gelsolin (5–10 nM) in the presence of low [Ca**^**2+**^**] (pCa 8.2) on HMM-induced F-actin sliding velocity *versus* filament length in the *in vitro* motility assay**. [Actin(monomer)]/[gelsolin]: 0.5 to 1. *A*, HMM incubation concentration 342.8 nM in the absence (*black dots*) and presence (*red dots*) of gelsolin. Full lines (with 95% confidence band; *dotted lines*) represent Equation 1 fitted to the data obtained in the control solution (orange; v^∞^ =1, [0.986, 1.014]; f= 0.118, [0.042, ∞]; mean, [95% CI]) and in the presence of gelsolin (blue; v^∞^ =0.759, [0.744, 0.775]; f = 0.0260 [0.0202, 0.042]; mean, [95% CI]). Data from six experiments. To be able to appropriately compare different experiments, the data in each individual experiment (30–80 filaments) were normalized to the average absolute velocity in the absence of gelsolin (8.42 [7.29, 9.06]; mean, [range]). Plots of data from individual experiments are presented in Fig. S2. *B*, HMM-induced F-actin sliding velocity with HMM added at 171.4 nM in the absence (*black dots*) and presence (*red dots*) of gelsolin. Full lines (with 95% confidence bands) represent Equation 1 fitted to the data obtained without gelsolin (orange; v^∞^=1.000, [ambiguous]; f = 0.781 [very wide] and in the presence of gelsolin (blue; v^∞^ =0.833 [0.821, 0.846]; f = 0.0572, [0.0467, 0.0786]; mean, [95% CI]). Note that the fits for the control data without gelsolin give very wide 95% CIs because of lack of data points indicating drop in velocity at short lengths. Note, further, the latter finding is consistent with a more substantial velocity reduction for short than long filaments after addition of gelsolin. Data from two different experiments, plotted as in A, with the average velocity in each individual experiment (61 and 106 filaments) in the absence of gelsolin, being 5.12 and 5.80 μm/s, respectively. Temperature: 25 to 27 °C. Data from gelsolin concentrations of 5 and 10 nM were pooled as no difference in effects were found in this range. HMM, heavy meromyosin.

To further investigate the interactions between gelsolin, actin, and myosin (HMM) in the presence of MgATP at low [Ca^2+^] (pCa 8.2), we analyzed gelsolin-induced changes in the filament sliding velocity in the *in vitro* motility assay. Generally, the average velocity decreased with increased gelsolin concentration in the range 1 to 10 nM. To gain insight into the mechanistic basis for this effect, we performed detailed analyses of the dependence of the sliding velocity on filament length using *in vitro* motility assays at HMM incubation concentration of 342.8 nM. In these analyses, we compared 5 or 10 nM gelsolin (both with similar effects; Fig. S2) to conditions without gelsolin. The results (Fig. 4) show that the presence of gelsolin reduces average velocity by i) reduced velocity of the longest filaments studied (reduction of v^∞^ in fit of Equation 1), ii) greater reduction in sliding velocity of short filaments (reduction of f in fit of Equation 1), and iii) reduction in the average length of the studied filaments.

We attribute the greater reduction in velocity for short filaments compared with long filaments to either of two mechanisms: (i) the gelsolin induced structural changes of the actin filament that explain the velocity reduction (*cf.* discussion below) are more marked at short distances from the gelsolin binding site, thus explaining greater effects on short filaments, (ii) gelsolin binding at the barbed end changes the structure of the entire actin filament to a similar degree, leading to reduced myosin association rate constant with reduced duty ratio for myosin heads binding all along the entire filament. In the latter case, one would expect greater reduction in velocity for short filaments because such filaments interact with fewer HMM molecules. If this (ii) is the key mechanism, one would also expect greater reduction in velocity (particularly for short filaments) at low (*e.g*., 171.4 nM) compared with high (*e.g.*, 342.8 nM) HMM surface density (*cf.* (58)). This was, however, not observed (Fig. 4*B*), arguing for the alternative idea of more extensive gelsolin-induced structural changes in F-actin close to the gelsolin binding site at the barbed end compared with more distant locations along the filament. The findings that the average velocity was reduced for the longest actin filaments at high motor surface density (Fig. 4*A*) is consistent with increased affinity between actin and myosin with reduced actomyosin detachment rate (59,60) in the presence of gelsolin (see further below).

### On the possibility of gelsolin-mediated displacement of phalloidin from the actin filaments

*In vitro* motility assay experiments performed in the presence of gelsolin resulted in the eventual loss of observable rhodamine phalloidin-labeled actin filaments from the motility assay surface as noted by tendency for a decrease in the number of filaments at times >60 s in Figure 3 and Figs. S1, *C*–*D* and S3*B*. These effects could be attributed to either of the following effects: (i) production of very short filaments either detaching from the surface or exhibiting too faint fluorescence (further aggravated by photobleaching) to be observed in epifluorescence microscopy or (ii) gelsolin-induced displacement of fluorescent phalloidin from actin filaments (61).

To investigate the latter possibility, we used covalently linked NHS–rhodamine instead of rhodamine phalloidin for fluorescence labeling of the actin filaments. The NHS–rhodamine–labeled actin filaments showed relatively weak fluorescence signal compared with those labeled with rhodamine phalloidin. In control motility assays, actin filaments whether labeled by NHS–rhodamine or rhodamine phalloidin behaved in apparently similar manner with long-term and durable motility (Fig. S3, *A* and *C*). Motility assays performed with NHS–rhodamine–labeled filaments in the presence of gelsolin (5 nM) and low calcium (pCa 8.2) resulted in slightly faster disappearance of the actin filaments (Fig. S3*D*) than in the case with rhodamine phalloidin (Fig. S3*B*). Therefore, these results suggest that gelsolin-based displacement of rhodamine phalloidin was not the basis for a reduced number of observable filaments with time in the presence of gelsolin. Rather, we favor the alternative explanation based on production of very small filament fragments that are either not observable because of faint fluorescence or that detach from the surface. This suggests an underestimation of the increase in the number of filaments with time in the *in vitro* motility assay in the presence of gelsolin (Figs. 2 and 3), and thus, underestimation of the severing efficiency cooperatively attributed to gelsolin- and myosin-induced forces.

### One-to-one relation and co-localization between gelsolin binding and severing in the absence of motor forces

We show above (Fig. 2 and Fig. S1) that high gelsolin and calcium concentrations induce rapid severing of the actin filaments under *in vitro* motility assay conditions. For practical purposes, to be able to follow the time course in those experiments, we had to limit either the gelsolin concentration or the Ca^2+^ concentration.

**Figure 5:**
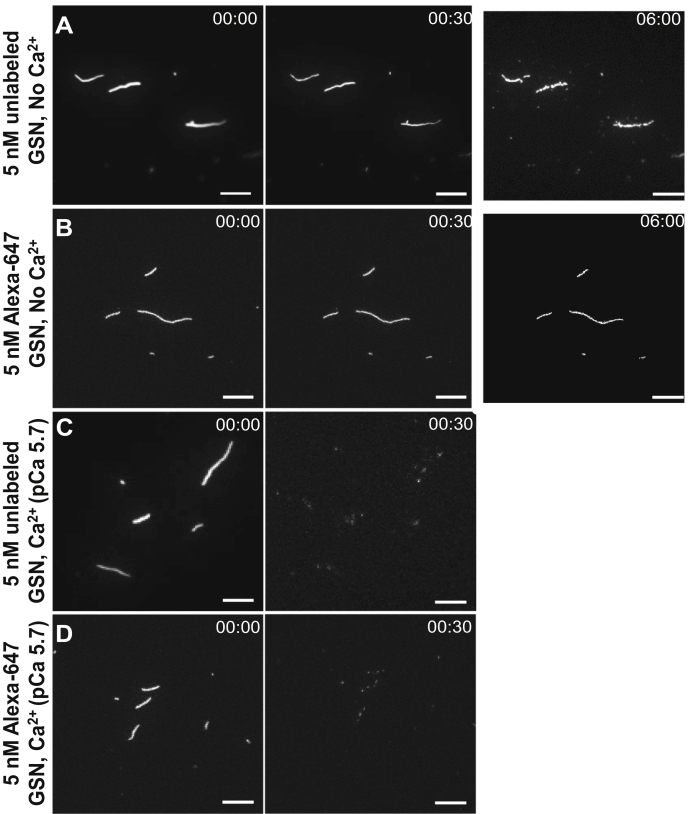
**TIRF microscopy images showing F-actin severing activity in the presence of 5 nM gelsolin either with or without calcium**. *A*, unlabeled gelsolin without calcium. *B*, Alexa-647-labeled gelsolin without calcium. *C*, unlabeled gelsolin, pCa 5.7. *D*, Alexa-647-labeled gelsolin, pCa 5.7. Preincubation with HMM at 34.2 nM before addition of actin filaments (rhodamine phalloidin labeled). Assay solution was added without MgATP, resulting in rigor actomyosin state keeping filaments stationary. Note, each image is an average of up to 100 subsequent image frames in a sequence. Image processing was done using ImageJ (Fiji, Ver. 1.53a). Time points are represented as min:sec. Scale bars, 10 μm. HMM, heavy meromyosin; TIRF, total internal reflection fluorescence. Temperature, 23 °C.

To study severing at high concentrations of both gelsolin (5 nM) and Ca^2+^ (pCa 5.7), we turned to TIRF microscopy to enable observation down to single fluorophores by limiting illumination to a ∼100 nm thick layer above the surface. Furthermore, to maintain high resolution and prevent detachment of very short actin filaments from the surface, as may occur in an *in vitro* motility assay (see above), the filaments were kept stationary on the surface by binding to HMM (added at 34.2 nM) without any added MgATP in a TIRF assay solution (see Materials and Methods). This facilitates observation of both individual actin filaments and Alexa-647-labeled gelsolin and therefore, of the gelsolin-binding and the associated severing pattern along F-actin. Using these conditions, we first noted a slow gelsolin-mediated fragmentation (half-life ∼5 min) of the filaments in the absence of Ca^2+^ (Fig. 5*A*), in agreement with bulk solution fluorescence spectroscopy data (Fig. 1). By increasing the [Ca^2+^] to pCa of 5.7 and with the same gelsolin concentration (5 nM) as above, the severing became very fast so that within 30 s, there were only tiny actin filament fragments left on the surface (Fig. 5, *C*–*D*) with similar behavior using Alexa-647-labeled and nonfluorescent gelsolin.

There is evidence (*e.g.*, (27, 45, 48)) that gelsolin, when bound to the barbed end of an actin filament, produces long-distance changes in filament structure. We therefore asked if gelsolin severing in the absence of HMM driven motion only occurs at the gelsolin binding site or if severing could occur at distant sites along the filament. To test this idea, we used Alexa-647-labeled gelsolin and TIRF microscopy–based single molecule studies. As shown above, we observed similar severing activity by the unmodified and the Alexa-647-labeled gelsolin whether using bulk depolymerization assays (Fig. 1) or TIRF microscopy–based observation of the filaments on a surface (Fig. 5).

**Figure 6:**
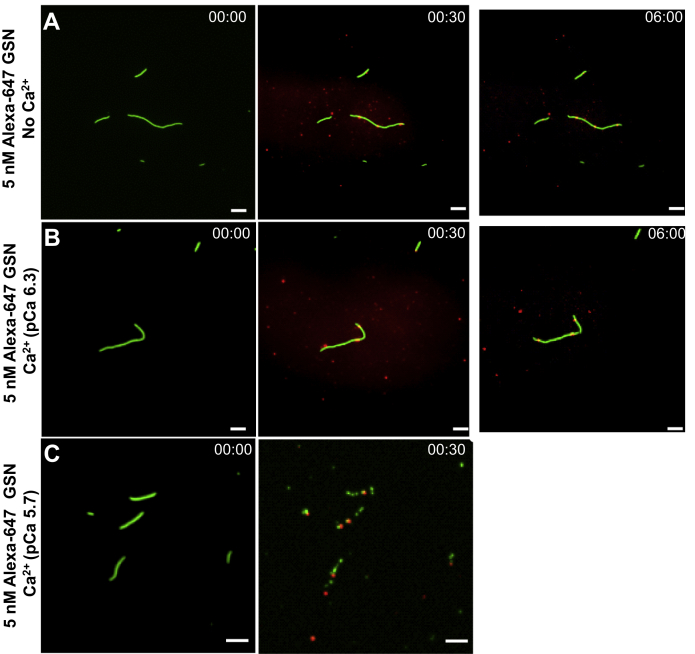
**TIRF microscopy images showing gelsolin binding and F-actin severing activity in the presence of 5 nM Alexa-647 gelsolin**. *A*, no calcium, same experiment and filaments as in Figure 5*B*. *B*, pCa 6.3, *C.*, pCa 5.7, same experiment, and filaments as in Figure 5*D*. Preincubation with HMM at 34.2 nM before addition of actin filaments (rhodamine phalloidin labeled). Assay solution was added without MgATP resulting in rigor actomyosin state. Note, images for the time point 00:00 are single channel images (rhodamine phalloidin; pseudocolored), whereas those in 01:00 and 06:00 are merged images of Alexa-647 and rhodamine phalloidin channels. Each image is an average of up to 100 subsequent images. Image processing was done using ImageJ (Fiji, Ver. 1.53a). Time points are represented in min:sec. Scale bars, 5 μm. Note, the 30 s frame in *C* shows the same filaments as in the 0 s frame, but the filaments have moved probably because some of the filament segments formed after cleavage were initially not directly linked *via* HMM to the surface, consistent with use of 10 times lower HMM incubation concentration (34.2 nM) than in the *in vitro* motility assay (342.8 nM). Temperature, 23 °C. HMM, heavy meromyosin; TIRF, total internal reflection fluorescence.

In the absence of added Ca^2+^(pCa > 9), we observed only limited binding of Alexa-647-labeled gelsolin molecules along the length of the actin filament and very limited filament severing activity (Fig. 6*A*; however, *cf.* Fig. 5, *A*–*B*). Of the 10 filaments observed, there were on average 1.7 gelsolin attachments with 0.5 cuts per filament during 9 min observation time. Addition of Ca^2+^ (pCa 7.3) (Fig. 6*B*) was ineffective in inducing actin filament severing in the absence of motor induced forces. However, at further increased [Ca^2+^] (pCa 5.7), rapid severing of actin filaments was observed, similar to the results obtained using unlabeled gelsolin (Fig. 5). Our use of TIRF microscopy to observe rhodamine phalloidin and Alexa-647 fluorescence under these conditions (Fig. 6*C*) showed binding of multiple gelsolins along the length of the actin filaments, and filament severing was primarily observed at the points of observed gelsolin attachment. As expected, gelsolin remained bound to one end of newly formed actin filament fragments after severing (Fig. 6). With 49 cuts observed on nine filaments, Alexa-647-labeled gelsolin was bound at 34 of the cuts. This gives a ratio of the number of gelsolin molecules to observed fragmentation points of 0.69, comparable to the Alexa-647/gelsolin labeling ratio of 0.67. These results support the view that gelsolin-mediated filament severing of stationary filaments is a local event because of conformational changes at the point of gelsolin binding. The results do not support ideas that severing takes place also at sites distant from the gelsolin binding site if the filaments are not propelled by HMM.

It is of interest to relate the results in Figures 5 and 6, without motility, to those in Figures 2 and 3 under *in vitro* motility assay conditions. However, partly because of the necessity to keep the severing rates within measurable ranges, direct comparisons should be treated with caution. Thus, the effect of gelsolin at pCa 5.7 was studied using gelsolin at 1 nM in Figure 2 but 5 nM under the stationary conditions in Figure 5, *C*–*D* and Figure 6*C*. Furthermore, the number of formed filament fragments during severing (and thereby the severing rate) is underestimated in the *in vitro* motility assays because epifluorescence illumination (used for practical reasons) fails to detect the smallest filament fragments. Additionally, the smallest actin filament fragments detach from the surface under *in vitro* motility assay conditions as indicated by later time points (>60 s in Figs. 2 and 3). Nevertheless, a semi-quantitative comparison for low [Ca^2+^] (pCa 8.2) and 5 nM gelsolin is possible (Fig. 3 *versus* Fig. 6). Thus, in the case without motility at low [Ca^2+^] (Fig. 6, *A*–*B*; pCa 6.3–9), virtually no cleavage upon addition of 5 nM gelsolin was observed within 60 s, whereas there was an appreciable increase in the number of filament fragments in the *in vitro* motility assay (Fig. 3) during this time at high HMM densities. The results suggest that gelsolin, when bound to actin at low [Ca^2+^] (*e.g.*, pCa 8.2, as used in Fig. 3) requires that motor-induced forces act on the filament to produce severing. In contrast, the severing of stationary filaments (Figs. 5 and 6) mediated by 5 nM gelsolin requires higher free [Ca^2+^] (characterized by a sharp threshold of pCa= 5.7–5.9), suggesting that the structural changes due to thermal fluctuations cannot be harnessed for gelsolin mediated cleavage at low [Ca^2+^]. However, at [Ca^2+^] above the threshold (pCa < 5.7), a slight further increase in Ca^2+^ concentration caused apparently complete fragmentation with rapid disappearance of the filaments also in the absence of motility.

### Gelsolin-binding to actin, myosin, or underlying surface during *in vitro* motility assays

**Figure 7:**
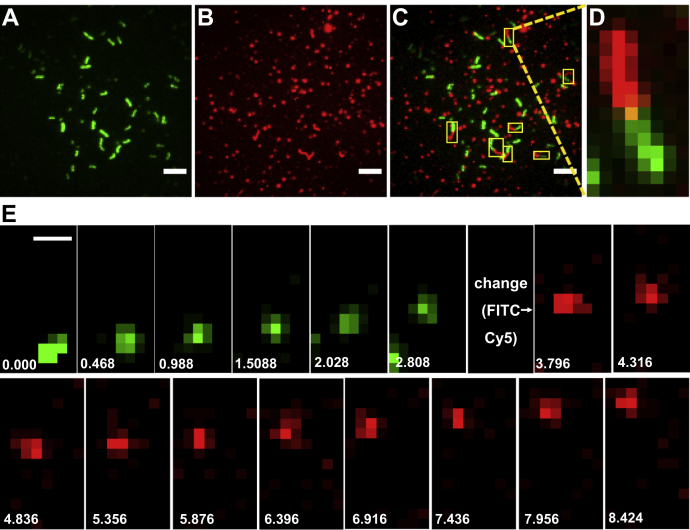
**Motility of actin filaments (*green*, Alexa-488 phalloidin labeled) with attached gelsolin (*red*, Alexa-647 labeled).** Conditions used in the assay, Ca^2+^(pCa 8.2), gelsolin (GSN; 5 nM), and HMM (342.8 nM). *A*, time projection image of background-subtracted video (summary of 57 subsequent frames acquired at rate of 20 s^−1^) showing actin filament fluorescence. *B*, time projection image of background-subtracted video (sum of sequence of 90 frames acquired at rate of 20 s^−1^) showing gelsolin fluorescence. Note that gelsolin can be bound to the actin filaments (motile trajectories observed) and nonspecifically to the surface (static spots; in majority). *C*, merged image of actin and gelsolin from *A* and *B* with motile actin filaments having bound gelsolin indicated by *yellow rectangles*. Note, visible double-colored filament trajectories. *D*, zoom in of one double-colored filament trajectory in *C* as indicated by *dashed lines*. *E*, Time-lapse sequence of the filament trajectory shown in *D* with filament moving from bottom to top. Video (see Video S1) was acquired as follows: first FITC filter cube was used to observe Alexa-488 phalloidin-labeled actin filament motility, followed by switch to Cy5 filter cube to observe Alexa-647-labeled gelsolin. Note, approximately 1 s delay associated with filter change. Video was recorded using TIRF microscopy and processed using ImageJ (Fiji, Ver. 1.53a). Background subtraction was done using the ImageJ function “Process/Subtract Background” with the parameter “Rolling ball radius” set to 10. Time points are represented in seconds. Scale bars, panels *A*–*C* = 5 μm, and panel E = 1 μm. Temperature, 23 °C. HMM, heavy meromyosin; TIRF, total internal reflection fluorescence.

To gain more insight into the gelsolin effects in the *in vitro* motility assay at low [Ca^2+^] (Figs. 3 and 4), we performed control experiments with *in vitro* motility assays under TIRF illumination in the presence of 5 nM Alexa-647-labeled gelsolin, using Alexa-488 phalloidin–labeled actin filaments. The results (Fig. 7) confirmed gelsolin binding to a substantial fraction of the HMM-propelled actin filaments (36.5%, n = 282 filaments studied from 10 image sequences). Interestingly, only those actin filaments that had bound gelsolin showed reduced velocity (Fig. S4). This corroborates the idea that the reduction in velocity is attributed to the bound gelsolin, and it is consistent with gelsolin-induced structural changes along the length of the filament. Considering the labeling efficiency of gelsolin with Alexa-647 (∼67%), the observed gelsolin labeling of 37% of the filaments in Figure 7 suggests that approximately 55% of all sliding filaments have bound gelsolin. Unfortunately, the locations of these gelsolin molecules along the filaments were not possible to determine using our set-up because of limited spatial resolution.

Nevertheless, binding of gelsolin to more than half of the filaments (55%) is consistent with the idea that binding of gelsolin contributes with as many cuts as the motor induced forces *per se*. This accords with the approximate doubling of the number of filaments after 60 s in the presence, as compared with the absence of gelsolin at the highest motor density in Figure 3. However, importantly, the lack of gelsolin effect at the lower motor densities in Figure 3 suggests that high motor induced forces, as those achieved at the highest motor density, are required to enable the gelsolin mediated cutting at low [Ca^2+^] (Figs. 3 and 7). This idea also accords with quite limited gelsolin-mediated severing by thermal fluctuations at low [Ca^2+^] (despite binding to actin) (Fig. 6).

It is also of interest to investigate the degree of gelsolin binding to the *in vitro* motility assay surface in the absence of actin. Surface-immobilized gelsolin molecules could serve as links that temporarily tether the actin filament to the underlying surface, thereby producing friction-like forces similar to those achieved using various actin binding proteins (*e.g.*, α-actinin) in so called frictional loading assays (reviewed in (62)). Such effects could potentially contribute to reduced sliding velocity (Fig. 4).

To investigate this possibility, we observed single molecule fluorescence of Alexa-647-labeled gelsolin after incubation of motility assay surfaces without actin filaments (Fig. S5). First, we found that the nonspecific surface adsorption of gelsolin was markedly increased at high compared with low [Ca^2+^] (Fig. S5, *A*–*D*; compare also Fig. S5, *E*–*F*). This is consistent with a more flexible gelsolin structure at micromolar Ca^2+^, because “soft proteins” are known (63) to have an increased propensity for surface adsorption. Furthermore, we noted that the surface adsorption was similar in the presence (Fig. S5, *A*–*D*) and absence (Fig. S5, *E*–*F*) of previous HMM incubation, at least at pCa 8.2. This argues for nonspecific binding of gelsolin to the underlying surface rather than to HMM. The limited nonspecific binding of gelsolin at the low Ca^2+^concentrations (Fig. S5, see also Fig. 7), used in the experiments in Figure 4, argues against major contributions from gelsolin-mediated surface friction to the reduction in sliding velocity of gelsolin-bound filaments. Moreover, gelsolin immobilized on the underlying surface (rather than on HMM) in these experiments is unlikely to interact with actin because HMM at its saturating density holds actin ∼50 nm away from the surface, which is much higher than the average gelsolin diameter (∼10 nm) (49, 64). Finally, the filament length dependence and the HMM surface density dependence of the gelsolin-induced reduction in sliding velocity (Fig. 4) argue against contribution from frictional forces because of interactions between the actin-bound gelsolin and the underlying surface. Any such effects would have been negligible for long filaments because of increasing motor-induced forces that overcome the friction caused by a single gelsolin molecule (*cf.* Fig. 7; Fig. S6). The effects would also have been more substantial at low HMM density because of lower overcoming motor forces. Neither of these effects were observed in Figure 4.

## Discussion

### Overall mechanistic interpretation

The results reflect a complex interplay between actin, HMM, [ATP], [Ca^2+^], and gelsolin. Myosin generates contractile, extensile, bending, and torsional forces (65, 66, 67) with shearing, buckling, and eventually severing of the actin filaments (25) even in the absence of actin-binding proteins such as cofilin and gelsolin. Myosin-driven actin filament fragmentation in the *in vitro* motility assay experiments occurs in the presence of nearly physiological (millimolar) concentrations of ATP (25). The motility experiments suggest that the gelsolin-mediated F-actin severing cooperates with the severing because of myosin motor function, in the sense that a given gelsolin concentration caused more extensive severing (greater increase in the number of filaments) in the presence of MgATP at increased HMM surface density. Conversely, increasing concentrations of gelsolin (even at low Ca^2+^-concentrations) led to increased actin filament fragmentation at a given HMM density (Fig. 3).

Before going into details of the myosin- and gelsolin-induced severing and the cooperativity between these processes, it is of relevance to briefly touch on a methodological issue. In the *in vitro* motility and TIRF microscopy assays, the actin filaments were generally labeled with rhodamine phalloidin or Alexa-488 phalloidin, whereas in the bulk depolymerization assays, pyrene-labeled actin was used. Some previous studies have emphasized the inhibitory role of phalloidin in depolymerization of actin filaments (9). However, phalloidin did not noticeably interfere with the gelsolin-mediated severing of actin filaments, in agreement with other previous reports (68, 69). In effect, the use of high concentration of gelsolin and Ca^2+^ resulted in extremely rapid severing of the phalloidin-labeled filaments both in motility assays and in TIRF microscopy–based single molecule studies. Furthermore, similar severing effects in the *in vitro* motility assays were observed for phalloidin labeled as for phalloidin-free filaments.

The mechanisms of gelsolin-induced actin filament severing have been investigated in appreciable detail previously (11), showing that sequential activation by Ca^2+^-binding to several sites on gelsolin lead to increased actin affinity with enhanced actin binding and eventual severing. The latter process has been described as opportunistic (11), taking advantage of thermal fluctuations of the actin monomers. Such fluctuations open for “insertion” of two of the six gelsolin domains between subsequent actin subunits along both protofilaments, with binding to subdomain 2 of one actin subunit and subdomain 1 of the neighboring subunit. The gelsolin binding sites (11, 70) are in close proximity to the myosin binding regions on the actin filament (71), but steric clashes between gelsolin and myosin along the filament cannot explain our observed effects on velocity (Fig. 4), because, generally, just one gelsolin molecule binds per filament (Fig. 7; Fig. S6).

The mechanisms for filament fragmentation caused by myosin motor forces have attracted fewer detailed studies than the mechanisms underlying gelsolin-mediated severing. However, the existence and importance of the phenomenon is well-known both from *in vitro* motility assays (*cf.* this study) and studies of living cells (28). Data in the literature (67, 72) suggest that actin filaments are highly resistant to breakage by extension but not to breakage by shearing forces caused by bending of the filament. This is also common experience from standard *in vitro* motility assays where filaments usually break on occasions when they make sharp turns. One may question if such sharp turns could be expected to occur in living cells where actin filaments move along myosin filaments with the myosin motors in straight lines rather than along randomly positioned HMM motor fragments on a flat surface as in the *in vitro* motility assay. However, the actin filaments are likely to switch between neighboring myosin filaments now and then in the cell because of similar mechanisms with thermal fluctuations of the leading filament tip, as in the *in vitro* motility assay. Additionally, bending may result from filament buckling (*cf.* (25, 73)). The likelihood of such buckling increases if bigger force-differences exist between neighboring attachment points to myosin along an actin filament and/or if the flexural rigidity (proportional to the persistence length) of the actin filament is reduced. The latter follows because the critical force of buckling is proportional to the persistence length (*cf.* (74)).

As mentioned above, both myosin and gelsolin seem to bind in similar regions on the actin subunits. Furthermore, the binding of either one molecule of gelsolin or one myosin molecule also seems to lead to structural changes in these regions (subdomains 1 and 2) propagating allosterically along the filament also to subunits without currently bound gelsolin or myosin. For the binding of a single gelsolin molecule at the barbed end of the actin filament, most studies seem to suggest that the structural changes propagate along the entire filament (27, 45, 48). For myosin, the extent of the propagation is not that clearly delineated, but also in this case, there is evidence for propagation a substantial distance away from the actual myosin binding site (57, 75, 76).

Now, one may consider possible mechanisms that could lead to cooperativity between effects of gelsolin and myosin. Of interest in this connection is previous evidence (77, 78) that increased tension in an actin filament increases gelsolin-mediated severing. Such an effect has the potential to contribute to the increased severing caused by gelsolin in the present *in vitro* motility assay experiments. First, the asynchronous action of different myosin motors along an actin filament, with different motors in different states and with different distortions at a given time, will temporarily produce local tension in the actin filament between different motors. Second, the bending motions produced by myosin action (*cf.* above) will cause increased tension on the “outer” perimeter of the curved filament. Third, in relation to the latter effect, it is straightforward to intuitively see how motor-induced bending of the filament would aid the opportunistic severing effect mentioned above with facilitated insertion of the critical gelsolin domains between neighboring actin subunits. Fourth, and finally, there is evidence to suggest (27, 44, 75, 79, 80) that myosin binding along the filament and gelsolin binding to the barbed end both modify the structure of the subdomains 1 and 2 in their partly overlapping gelsolin-binding and myosin-binding regions on actin (11, 71), even outside the actual actin subunit where myosin and gelsolin bind. Therefore, it is not far-fetched that the structural changes caused by such binding by one of the proteins could modulate the affinity of the other protein along the entire filament. Indeed, our data in Figure 4, with reduced velocity for filament at all lengths, are consistent with increased actin affinity for myosin when gelsolin binds to the barbed end. This follows because the sliding velocity (at least with large number of interacting motors as with long filaments) is generally believed to be directly proportional to the cross-bridge detachment rate constant from strongly bound states (59, 60). A reduction in the latter rate constant is consistent with increased actomyosin affinity. The velocity reduction could, however, also be because of increased affinity in a weakly bound actomyosin state as recently suggested to be the mechanism behind velocity reduction by the small molecule myosin inhibitor blebbistatin (81). Finally, an increased actomyosin affinity would contribute to increased severing by motor induced forces due to increased propensity for local buckling of the filament (see above).

Strikingly, our results suggest that structural changes along the actin filament upon gelsolin binding are produced both at micromolar and nanomolar free [Ca^2+^]. This is partially consistent with the results in (47), where it was proposed that the degree of calcium binding to gelsolin at very low [Ca^2+^] (pCa 7.3–8.0) causes structural change that unlatches the closed structure of gelsolin (47). The necessity for simultaneous myosin motor activity to effectively harness the gelsolin effects at low [Ca^2+^] follows from results in Figures 1, 3, and 6, and the possible molecular mechanisms are discussed in some detail above. These mechanisms lead to appreciably increased filament severing upon gelsolin addition during myosin-induced sliding at high HMM density (Fig. 3). In contrast, very limited severing occurs in the absence of such forces (Figs. 1 and 6) or when the forces are low (low HMM densities in Fig. 3).

### Implications for cellular physiology

The intrinsic activities of gelsolin and myosin can provide control of severing and contractility/fragmentation, respectively in the cellular environment (11, 28, 82). However, cooperative severing due to the forces induced by the simultaneous action of gelsolin and myosin on the actin filament would result in more efficient severing than by either of the proteins alone and would also produce a mixture of gelsolin-capped and gelsolin-free filament fragments. The results suggest that fine-tuning of actin filament contractility/disassembly in response to the differences in the distribution/concentration of gelsolin, myosin, and [Ca^2+^], as well as the mechanical state of the actin filaments in different cellular compartments may reflect the cooperative effects of gelsolin and myosin. In smooth muscles, contraction is initiated upon increased [Ca^2+^] by the phosphorylation of myosin II by Ca^2+^/calmodulin-dependent myosin light chain kinase. In contrast, in certain nonmuscle cells demonstrated for fibroblasts and hepatic stellate cells, myosin II activation and contractile force generation is independent of changes in [Ca^2+^] and occurs at low cytosolic levels of the divalent cation (83, 84). Under these low [Ca^2+^] conditions, when gelsolin is expected to be inefficient in severing on its own, it can cooperatively harness the myosin-induced forces to facilitate filament fragmentation. On the other hand, upon elevation of the cytosolic calcium levels, nonmuscle myosin IIA and gelsolin associate to collagen adhesion sites where their functional interdependence promotes actin reorganization required for the integrin-dependent phagocytosis of collagen fibrils in mouse fibroblasts (85, 86). In high [Ca^2+^] conditions, the cooperative severing could provide extremely efficient fragmentation and depolymerization in processes that rely on dynamic actin remodeling.

Of further interest, recently gelsolin was implicated in biomechanical stress-induced mechanotransduction in cardiac cells (82). The severing activity of gelsolin was found to be stimulated by mechanical loading in dilated cardiomyopathy, whereas unloading by left-ventricular assist devices therapy restored severing. Consistent with this, pressure-overload stimulated the severing of cytoskeletal actin filaments by gelsolin in mouse cardiomyocytes, in contrast, in gelsolin KO mice aberrant cytoskeletal remodeling, and heart failure was prevented.

### Conclusions

In conclusion, we have found strong evidence for cooperative effects between gelsolin-induced changes in the actin filaments and changes due to myosin motor activity, mainly in the following respects. The gelsolin-induced changes in filament structure increase the susceptibility of the filament to myosin-generated forces, possibly *via* increased actin–myosin affinity, which would also account for reduced myosin propelled actin filament velocity. Moreover, the local tension along the actin filament (particularly because of bending of the filament) that is produced by myosin motor activity facilitates the opportunistic cleavage by gelsolin. The results are consistent with the idea of long-range structural changes in the actin filaments in response to gelsolin binding. However, in view of greater gelsolin-induced reduction in velocity of short filaments, our results suggest that the structural changes are more prominent at shorter distances from the gelsolin capped barbed end. Finally, our results suggest that appreciably enhanced severing of actin filaments may be achieved by combined actions of gelsolin and myosin of possible physiological relevance (*e.g.*, (82, 83, 84, 85, 86)).

## Experimental procedures

### Ethical statement

Animal handling and experiments were approved by the Regional Ethical Committee for Animal Experiments in Linköping, Sweden (reference number 73-14).

### Chemicals

Rhodamine phalloidin (phalloidin conjugated to tetramethylrhodamine isothiocyanate), N-hydroxy-succinimidyl rhodamine (NHS–rhodamine), Alexa Fluor 488 Phalloidin (Alexa-488 phalloidin), and Alexa Fluor 647 C_2_ Maleimide (Alexa-647), N-(1-Pyrenyl) iodoacetamide (pyrene) were purchased from Thermo Fisher Scientific. Bovine serum albumin (BSA [standard purity], BSA [high purity, (87)]) and all other analytical grade chemicals and reagents used in this study were purchased from Sigma-Aldrich Sweden AB (now Merck), unless otherwise stated.

### Protein preparations

Actin was purified from leg and back muscles of rabbit using the acetone powder method and was quickly snap frozen in liquid nitrogen (88), followed by storage at −80 °C. Myosin II was isolated from rabbit leg muscles and subsequently digested with N-R-tosyl-L-lysine chloromethyl ketone–treated α-chymotrypsin to obtain HMM (89), which was frozen in the presence of 2 mg/ml sucrose and stored at −80 °C. F-actin (0.25 mg/ml) was labeled with either rhodamine phalloidin or Alexa-488 Phalloidin at a molar ratio of 1:1.5 (actin:phalloidin) in 10 mM 4-morpholinepropane-sulfonic acid buffer at pH 7.0 containing 60 mM KCl, 2 mM MgCl_2_, 0.1 mM EGTA, and 3 mM sodium azide (NaN_3_). NHS–rhodamine labeling of F-actin was performed according to the manufacturer's instructions. Protein concentration (HMM, actin) was measured by UV absorbance spectroscopy, whereas protein purity was confirmed by SDS-PAGE.

The plasmid DNA of His-tagged recombinant full-length human cytoplasmic gelsolin (pET21 day (+)) was transformed into *E. coli* BL21 (DE3) cells (21, 90). A fresh colony of *E. coli* was grown in LB broth at 37 °C until the OD_600_ reached 0.6 to 0.8 and then induced with 1 mM isopropyl β-D-1-thiogalactopyranoside overnight at 25 °C. The cells were collected by centrifugation (6000*g*, 5 min, 4 °C, Sigma 4-16KS tabletop centrifuge), lysed in lysis buffer (5 mM Tris, 300 mM NaCl, 5 mM imidazole, 1 mM ATP, 1 mM phenylmethylsulfonylfluoride, 7 mM β-mercaptoethanol, 30 mg/ml DNase with protease inhibitor cocktail [P8465, Sigma-Aldrich] [pH 8.0]) and then sonicated and ultracentrifuged (440,000*g*, 35 min, 4 °C; MLA80 rotor, Beckman Optima MAX-TL). The supernatant was applied to a Ni-NTA column (Machery-Nagel), washed with lysis buffer, and eluted with 250 mM imidazole in lysis buffer. Fractions containing gelsolin were dialyzed (20 mM Tris, 1 mM EGTA [pH 8.0]) and further purified on a Source 15Q anion exchange column (GE Healthcare) with the application of 50 ml buffer I (20 mM Tris, 20 mM NaCl, 1 mM EGTA [pH 8.0]), 50 ml buffer II (10 mM Tris, 0.1 mM EGTA [pH 8.0]), 50 ml buffer III (20 mM Tris, 2 mM CaCl_2_ [pH 8.0]), and a 100 ml linear gradient of buffer III and buffer IV (20 mM Tris, 1 M NaCl, 0.1 mM EGTA [pH 8.0]). The gelsolin-containing fractions were dialyzed (5 mM HEPES, 50 mM NaCl, 0.1 mM EGTA [pH 8.0]), and gel filtered on a Superdex 200 column (GE Healthcare) equilibrated with dialysis buffer. Purified gelsolin was collected, concentrated (Vivaspin10 K cut-off tubes [Sartorius]; 3000 g, 4 °C, 4–16KS tabletop centrifuge), snap frozen in liquid nitrogen, and stored at −80 °C. The protein concentration was measured by spectrophotometry (ε_280_ = 1.29 ml mg^−1^ cm^−1^). Gelsolin was labeled by Alexa-647 (8-fold molar excess) for 2 h at room temperature. The unbound dye was removed by using a PD-10 column (GE Healthcare). The final protein and probe concentrations were determined spectrophotometrically. The molar ratio of the bound probe to gelsolin was 0.67.

### Dilution-induced depolymerization assays

First, 50 μM MgCl_2_ and 200 μM EGTA were added to 1.15 μM G-actin to replace the actin bound Ca^2+^ to Mg^2+^ (final concentrations). The Mg^2+^-G-actin in buffer A (4 mM Tris, 0.1 mM CaCl_2_, 0.2 mM ATP, 0.005% NaN_3_, 0.5 mM β-mercaptoethanol [pH 7.8]) at 1 μM concentration (50% pyrene labeled) was polymerized overnight by adding 2 mM MgCl_2_ and 100 mM KCl (final concentrations). The F-actin sample was then diluted to 20 nM with Ca^2+^-free polymerization buffer (4 mM Tris, 0.2 mM ATP, 0.005% NaN_3_, 0.5 mM β-mercaptoethanol, 2 mM MgCl_2_, 100 mM KCl [pH 7.8]) supplemented with 100 μM CaCl_2_ or 1 mM EGTA to obtain the desired Ca^2+^ concentrations. Depolymerization rates were estimated by linear fitting of the normalized pyrene transient curves (first 500 s in the presence of 1 mM EGTA or 40 s in the presence of 100 μM CaCl_2_).

### *In vitro* motility assay

*In vitro* motility experiments were performed at 25 to 27 °C on glass surfaces silanized with trimethylchlorosilane (TMCS) as previously described (91, 92). During a given experiment, the temperature was kept constant to within 1 to 2 °C. Briefly, silanization was performed as follows; first glass cover-slips (60 × 24 mm^2^, #0, Menzel Gläser) were cleaned with piranha solution (H_2_SO_4_ and 30% H_2_O_2_ at 7:3 ratio; *note that piranha solution is highly corrosive, acidic and reacts violently with organic materials. Therefore, follow appropriate safety precautions*) at 80 °C for 5 min followed by sequential washing with H_2_O (thrice), methanol, acetone, and chloroform. Cleaned glass coverslips were then functionalized with 5% TMCS in chloroform for 2 min and washed with chloroform. Thus, functionalized surfaces were dried under a dry N_2_ gas stream and stored under ambient conditions (Petri dishes sealed with parafilm) (92).

Motility assays were performed using the following buffer solutions: (1) Low ionic strength solution (LISS): 1 mM MgCl_2_, 10 mM 4-morpholinepropane-sulfonic acid, 0.1 mM K_2_EGTA, pH 7.4. (2) L65: LISS containing 50 mM KCl and 10 mM dithiothreitol (DTT). (3) A60 Assay solution of 60 mM ionic strength, containing 1 mM MgATP, 10 mM DTT, and 45 mM KCl added to LISS solution supplemented with an antibleaching mixture containing 3 mg/ml glucose, 0.1 mg/ml glucose oxidase, 0.02 mg/ml catalase, and an ATP regenerating system containing 2.5 mM creatine phosphate and 0.2 mg/ml creatine phosphokinase. Flow cells were assembled using double-sided adhesive tape to form a fluid chamber between a nonfunctionalized small cover slip (20 × 20 mm^2^) and a TMCS-functionalized large cover slip (60 × 24 mm^2^). In a typical motility assay, the flow cell was infused sequentially with the following solutions: (1) HMM (342.8 nM; 120 μg/ml) diluted in L65, for 5 min, (2) BSA (standard purity, 1 mg/ml) in L65 for 2 min, (3) Wash (L65, once), (4) F-actin (0.25 μg/ml) labeled with rhodamine phalloidin or Alexa-488 phalloidin in L65, (5) Wash (L65, thrice), (6) A60 (either cold, which is incubated for 2 min or prewarmed). For gelsolin-mediated F-actin severing experiments, gelsolin (1–10 nM) and calcium (details below) were added into the A60 assay solution, without changing the concentration of any other reagents from the values above. The fluorescence images of F-actin sliding on HMM were captured using an inverted fluorescence microscope (AxioObserver D1, Zeiss) with 63× plan apochromat objective (Zeiss: 1.4 N A). The image sequences were recorded using a digital CCD camera (C4742-95, Orca-ER, Hamamatsu Photonics) or an EM-CCD camera (C9100, Hamamatsu Photonics) using HCImage software. The resolution of the recorded images, at an overall image size of 512 × 512 pixels, was 0.198 μm^2^/pixel. The frame rates used were 5 s^−1^. The image sequences were analyzed using MatLab software (MatLab R2017a; MathWorks) to obtain filament sliding velocities (93, 94). Lengths of sliding and stationary actin filaments were obtained from calibrated intensity data as described previously (56).

Assay solutions with different concentrations of calculated free Ca^2+^ were prepared for the experiments with and without gelsolin by modifying the above mentioned A60 solution as given in parentheses in the following: 0 nM free Ca^2+^ (addition of 100 μM EGTA, no added CaCl_2_), 6.8 nM free Ca^2+^ (pCa 8.2, 100 μM EGTA, 10 μM CaCl_2_), 1.1 μM free Ca^2+^(pCa 5.9, 100 μM EGTA, 97 μM CaCl_2_), and 1.9 μM free Ca^2+^ (pCa 5.7, 100 μM EGTA, 100 μM CaCl_2_). The free Ca^2+^ concentrations were calculated by the Maxchelator program, version WEBMAXC STANDARD, that is typically used to determine the free metal concentration present in the solution in the presence of chelators (95, 96). Above, and elsewhere in this article, the calculated free [Ca^2+^] is also expressed as the negative decadic logarithm, pCa = − Log_10_ [Ca^2+^].

### TIRF assay

TIRF assay solution, pH 7.4 was prepared to contain (final concentrations): 2 mM Trolox, 2 mM Cyclooctatetraene (COT), 2 mM 4-Nitrobenzyl alcohol (NBA), 10 mM DTT, 45 mM KCl, 7.2 mg/ml glucose, 3 U/ml pyranose oxidase, 0.01 mg/ml catalase, 2.5 mM creatine phosphate, and 0.2 mg/ml creatine phosphokinase in LISS (97). Initially, 100 mM Trolox stock was prepared in methanol, followed by dilution in LISS, subsequent filtering through a 0.2 μm filter and exposure to UV light (254 nm, 120,000 μJ/cm^2^) for 15 min to form Trolox-Quinone. The Trolox-Trolox/Trolox-Quinone mixture prepared in LISS was degassed before use. For further details, see (97).

The TIRF assay was performed by first adsorbing HMM (34.2 nM incubation concentration for assays without motility) onto TMCS-derivatized glass surfaces (5 min). Subsequently, the flow-cell was infused sequentially with 1 mg/ml BSA (2 min; high purity, see under Chemicals above), wash buffer L65, rhodamine/Alexa-488 phalloidin-labeled F-actin (5 nM, 2 min), and gelsolin (5 nM, labeled with Alexa-647) with varying concentrations of free calcium (see above) as desired. All TIRF assay experiments were performed at a stable temperature (23 °C ± 1 deg. C), using an objective heater (97). Time-lapse movies were acquired with an exposure time of 50 ms with 20 s^−1^ frame rate. An objective-based TIRF microscopy system was built in house using a Nikon TIRF 60× objective (NA = 1.49), a Nikon Eclipse TE300 inverted microscope, and an Andor iXon Ultra 897 EMCCD camera. Furthermore, we used a 642 nm diode laser (Melles Griot, 56RCS/S2799, OEM diode laser, 45 mW) as described elsewhere (97), along with addition of a blue laser (Changchun New Industries Optoelectronics Tech. Co, Ltd, Blue Solid State Laser, MLL473, 473 nm, 50 mW) enabling dual color illumination of Alexa-488 phalloidin–labeled actin and Alexa-647-labeled gelsolin during *in vitro* motility assay experiments. For these *in vitro* motility assay experiments the HMM incubation concentration was increased to 343 nM, in order to saturate the surface with HMM, and 1 mM MgATP was included in the TIRF assay solution.

The simultaneous addition of NBA, COT, and Trolox, to improve dye photophysical properties in the single molecule TIRF assay, reduced the actin filament sliding velocity, whereas Trolox alone had negligible effects (compare Fig. S4*A* to Fig. S4*B*). Importantly, however, the effects of gelsolin on velocity were similar whether only Trolox or both Trolox, COT and NBA were present (compare Fig. S4*A* to Fig. S4*B*), justifying the use of all components to ensure optimal image quality.

### Statistical analysis and analysis of velocity *versus* filament length plots

Data were analyzed using MatLab software as described above, and the subsequent nonlinear and linear curve fittings and statistical analyses were performed using Graphpad Prism (version 8.2.1, Graphpad software). Unless otherwise stated, data are given as mean ± 95% confidence interval. In statistical analyses, we assume that data are sampled from approximately normal distributions. Whereas, we cannot fully exclude deviations from this assumption the present size of our data set does not allow unequivocal assessment of this possibility. Under our assumptions, nonoverlapping 95% confidence intervals between groups indicate (to fair degree of approximation) statistically significant differences between mean values (*p* < 0.05).

The relationship between actin filament length (l) and sliding velocity (v_f_) in the *in vitro* motility assay was fitted by the semiempirical equation (58, 98).

(1) $${\text{v}}_{\text{f}}={\text{v}}^{\infty }\left(1-{\left(1-\text{f}\right)}^{\rho \text{dl}}\right)$$

Here v^∞^ is the velocity at infinite filament length, f was originally (58) defined as the actin–myosin duty ratio, ρ as the myosin head density on the surface, and d as the width of a band around the actin filament where the myosin heads are in reach for binding to actin. Here, we do not interpret the parameters f, ρ, and d strictly according to the initial definition because of complexities revealed in recent studies using *in vitro* motility assays at high motor surface densities (98, 99). However, nevertheless, the equation is useful to describe the characteristics of the velocity-length plot. Thus, a low value of v^∞^ denotes a low average velocity for long filaments whereas a low value of f denotes lower velocities for short than for long filaments if ρ and d are held constant. Here, we assumed that d is 30 nm, whereas ρ is taken as 5000 μm^−2^ and 2500 μm^−2^ after HMM incubation at concentrations of 343 nM and 171 nM, respectively. The latter values are in approximate agreement with data in the literature for TMCS-derivatized surfaces (*cf.* (100)) as used here. However, importantly, the exact numerical values used for ρ and d are not critical for the interpretations of changes in v^∞^ and f upon addition of gelsolin.

## Data availability

The data from this study are either contained within the manuscript and the supporting information or can be shared upon request to the corresponding author (Alf Månsson, alf.mansson@lnu.se).

## Conflict of interest

The authors declare that they have no conflicts of interest with the contents of this article.
