# Supplementary material for: Myosin and gelsolin cooperate in actin filament severing and actomyosin motor activity
Source: J Biol Chem. 2020 Dec 17;296:100181. doi: 10.1074/jbc.RA120.015863 (PMC7948409; doi:10.1074/jbc.RA120.015863)
Supplement: Figures S1–S6 [file mmc1.docx]

**Supporting information**

**Myosin and gelsolin cooperate in actin filament severing and actomyosin motor activity**

by

Venukumar Vemula^1^, Tamás Huber^2^, Marko Usaj^1^, Beáta Bugyi^2^ & Alf Månsson^1^.

^1^ Linnaeus University, Dept. of Chemistry and Biomedical Sciences, Universitetskajen, 391 82 Kalmar.

^2^ University of Pécs, Medical School, Department of Biophysics, Szigeti str. 12, Pécs, H-7624, Hungary

*
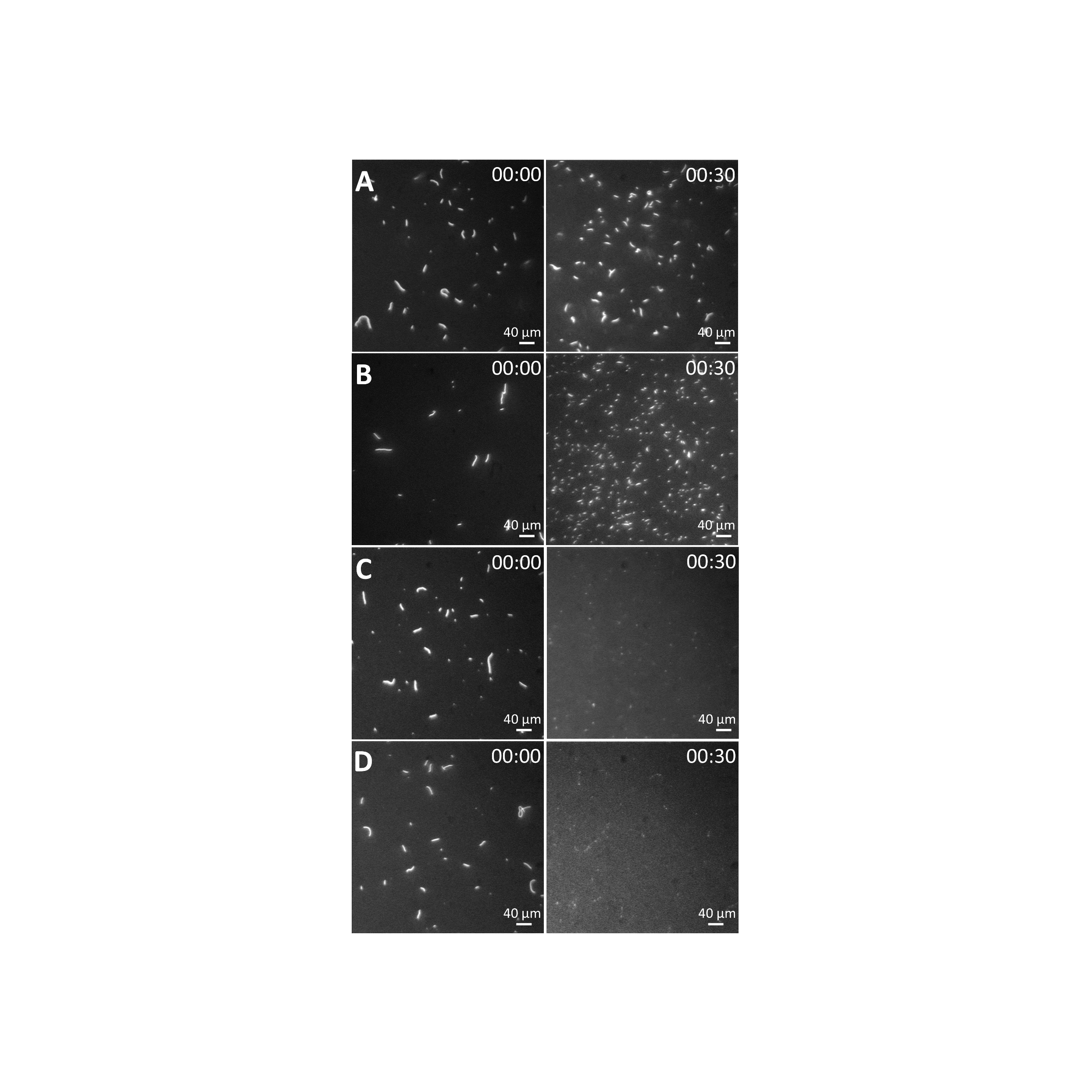
*

***Figure S1. Fluorescence microscopy images of rhodamine phalloidin labelled actin filaments gliding over HMM in an in vitro motility assay using A60 assay buffer.*** *A) Control assay without gelsolin, B) 5 nM Gelsolin in the presence of Ca^2+^ (pCa 8.2), C) 5 nM Gelsolin in the presence of Ca^2+^ (pCa 7.2), D) 5 nM Gelsolin in the presence of Ca^2+^ (pCa 5.7). Time points represented in minutes:seconds.*

***Figure S2. Experimental raw data for main Fig. 4A.*** *These data from six different experiments (A-F) are all replotted together in main Fig. 4A after normalization to the average velocity in the control solution in each given experiment. Black: control solution without added gelsolin. Red: after addition of gelsolin. All experiments performed using 5 nM gelsolin except B and D where the gelsolin concentration was 10 nM. More experimental details in the main paper. Note, all experiments show a tendency for reduced velocity at all filament length but in all cases the effect is greatest for filaments of short length. Further, note, no consistent difference between the effects of 5 and 10 nM gelsolin.*

*
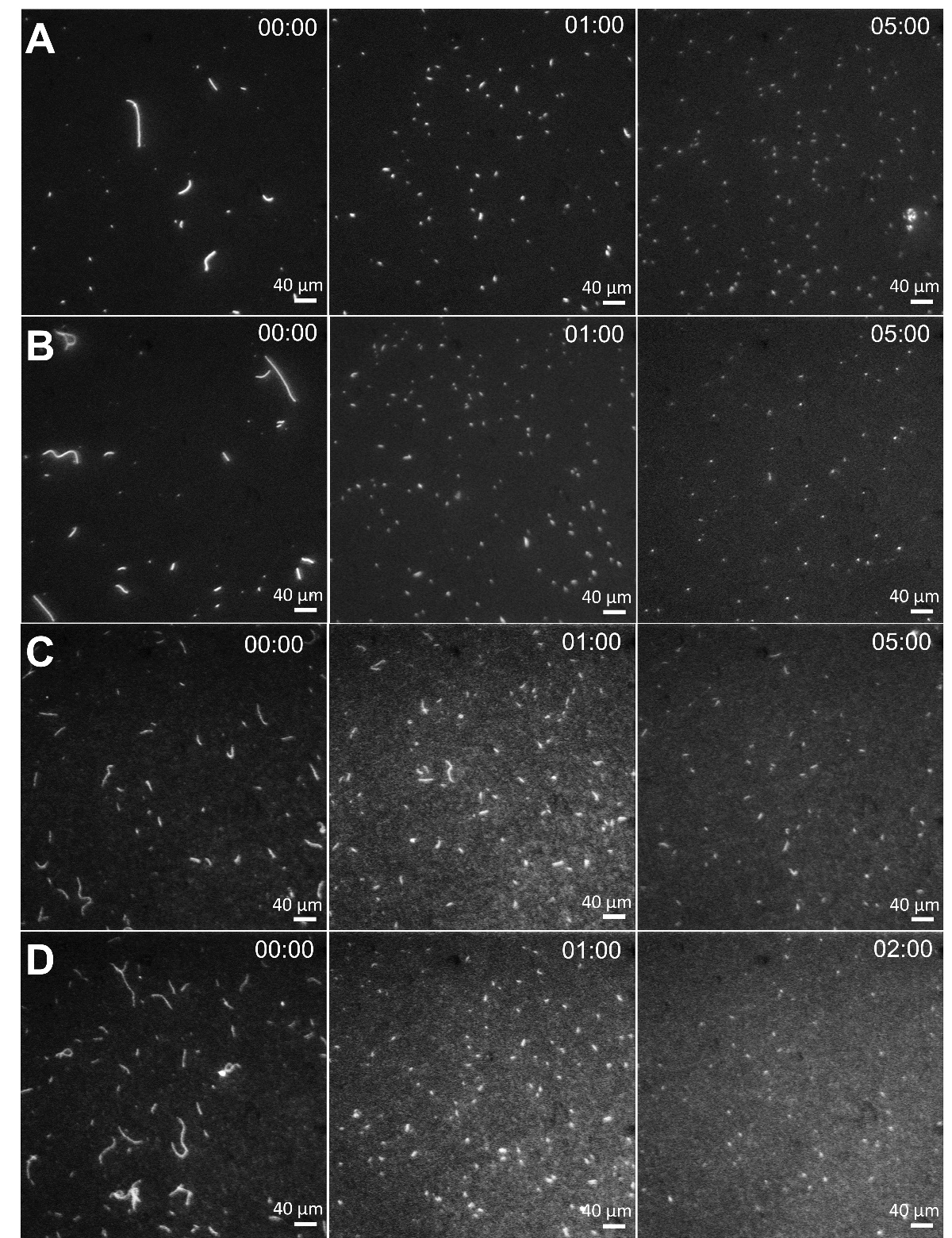
*

***Figure S3. Fluorescence microscopy images of actin filaments gliding over HMM in an in vitro motility assay in A60 assay solution.*** *A) Control assay showing rhodamine phalloidin labelled actin filaments. B) Rhodamine phalloidin labelled actin filaments in the presence of 5 nM gelsolin, 100 µM EGTA and low [Ca^2+^] (pCa 8.2). C) Control assay showing NHS-rhodamine labelled actin filaments D) NHS-rhodamine labelled actin filaments in the presence of 5 nM gelsolin and low [Ca^2+^] (pCa 8.2). Time points represented in minutes:seconds.*

***Figure S4. Effects of gelsolin on sliding velocities of actin filaments in the in vitro motility assay.*** *A) Motility assay either without (ACTIN) or with gelsolin (ACTIN+gelsolin) added to the flow cell. Motility assay solution A60, containing Trolox (2 mM) to minimize photophysical effects in single molecule fluorescence. B) Motility assay velocities from a flow cell without added gelsolin (ACTIN) is compared to motility of gelsolin-bound (ACTIN+gelsolin) and gelsolin free (ACTIN W/O GSN) filaments in a flow cell with added gelsolin. The gelsolin-binding to the filaments was identified using TIRF microscopy and Alexa-647 labelled gelsolin. Motility assay solution A60 containing both Trolox (2 mM), NBA (2 mM), and COT (2 mM) for radically minimizing photophysical effects in single molecule fluorescence. The concentration of other reagents used in the assay: HMM: 342 nM, F-actin: 5 nM, Calcium (pCa 8.2) and GSN:5 nM. Note that the additional components NBA and COT do not noticeably alter the observed effects of gelsolin although the maximum sliding velocity is reduced by the addition of these components.*


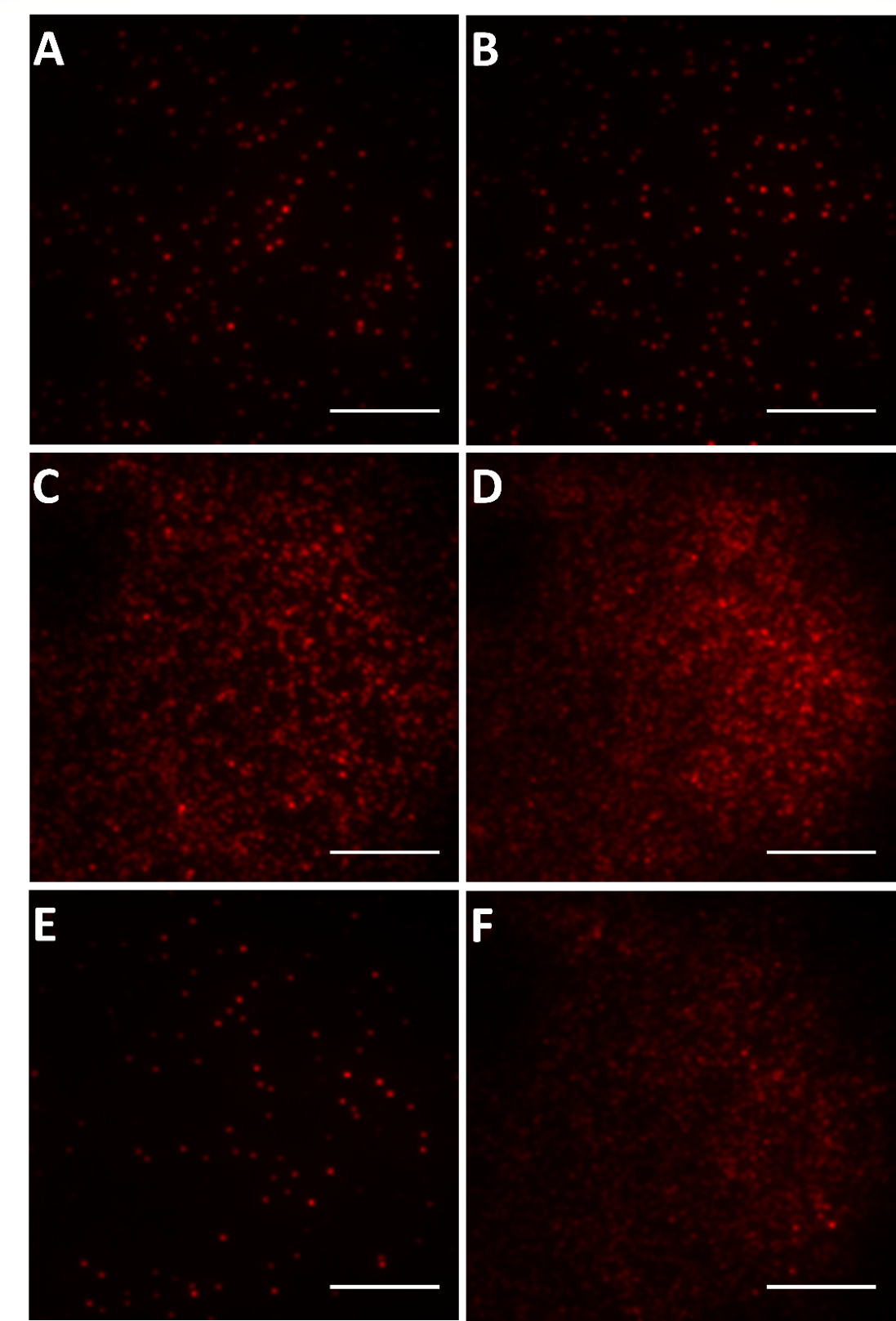


***Figure S5. TIRF Microscopy images showing average intensity Z-projection plots (averaging of 290 frames acquired at rate of 20 s^-1^) of Alexa-647 labelled gelsolin bound to TMCS surface at different calcium concentrations with (342.8 nM) or without HMM preincubation.*** *A) HMM, No Calcium B) HMM, pCa 8.2 C) HMM, pCa 5.7 D) HMM, pCa 3.5 E) No HMM, pCa 8.2 F) No HMM, pCa 3.5. Note, images were acquired using filter Cy5 (suitable for Alexa647 fluorophore) by TIRF microscopy and were processed using ImageJ (Fiji, Ver. 1.53a). Each image is an average of 290 subsequent images. Image scale, 10 µm. Note, that gelsolin exhibits appreciably increased non-specific surface binding in the presence of increasing calcium concentrations, consistent with the transformation of gelsolin to a more flexible (“soft”) protein [1] at increased [Ca^2+^].*

**
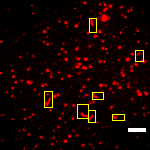
****A) C)**

**B)**

***Figure S6. Evidence for one gelsolin molecule per motile actin filament.* *A)*** *Summary of 90 subsequent background-subtracted images in a video sequence at 20 s^-1^ frame rate of Alexa647 labelled gelsolin. Same data as in main Fig. 7. Note that gelsolin moves only if bound to motile actin filaments (motile trajectories observed in yellow rectangles). Otherwise gelsolin is bound non-specifically to the surface or to non-motile actin filaments (static spots, here in majority). Scale bar, 5 µm. B) Examples of characteristic single molecule one-step Alexa-647 photobleaching of static gelsolin to determine single fluorophore fluorescence intensity values. C) Average fluorescence intensities of different gelsolin subgroups. The first group to the left for single molecule photobleaching include data from the records in B. Note, variability related to differences in illumination over the field of view and possibly different photophysical environments of the fluorophores, e.g. with the gelsolin being adsorbed to the surface with the Alexa-647 moiety touching the surface or projecting out into the solution. Note that all motile gelsolin molecules studied share the lowest average fluorescence intensity observed suggesting that only a single gelsolin molecule is attached to each motile actin filament studied. In contrast, the static gelsolins can be found in three clearly separated groups of intensity values close to multiples of the mean fluorescence intensity for the group of faintest fluorescence. We interpret the three groups as corresponding to single molecules (majority) or aggregates of two and three molecules. Data given as mean ± 95% CIs.*

**Supporting References**

1. Månsson, A., et al., *In vitro assays of molecular motors - impact of motor-surface interactions.* Frontiers in Bioscience, 2008. **13**(May 1): p. 5732-5754.
